# High levels of fasting glucose and glycosylated hemoglobin values are associated with hyperfiltration in a Spanish prediabetes cohort. The PREDAPS Study

**DOI:** 10.1371/journal.pone.0222848

**Published:** 2019-09-19

**Authors:** Antonio Rodríguez-Poncelas, Josep Franch-Nadal, Gabriel Coll-de Tuero, Manel Mata-Cases, Margarita Alonso-Fernández, Teresa Mur-Marti, Antonio Ruiz, Carolina Giraldez-García, Enrique Regidor

**Affiliations:** 1 METHARISC Group, USR Girona, IDIAP Gol i Gorina, Girona, Spain; 2 Grup de Recerca Epidemiològica en Diabetis des de l´Atenció Primària (DAP_CAT) Jordi Gol, Barcelona, Spain; 3 RedGDPS Foundation, Madrid, Spain; 4 USR Barcelona ciutat–IDIAP Jordi Gol, Barcelona, Spain; 5 CIBER Diabetes y Enfermedades Metabólicas Asociadas (CIBERDEM), Madrid, Spain; 6 Departament de Medicina, Universitat de Barcelona, Barcelona, Spain; 7 Departamento de Ciencias Médicas, Universitat de Girona, Girona, Spain; 8 Centro de Salud La Ería, Asturias, Spain; 9 Departamento de Medicina Preventiva y Salud Publica, Universidad de Oviedo, Asturias, Spain; 10 Mutua Terrassa, Barcelona, Spain; 11 Centro de Salud Universitario Pinto, Madrid, Spain; 12 Hospital Universitario Infanta Elena, Madrid, Spain; 13 Departamento de Salud Pública y Materno-Infantil, Facultad de Medicina, Universidad Complutense de Madrid, Madrid, Spain; 14 CIBER Epidemiología y Salud Pública (CIBERESP), Madrid, Spain; 15 Instituto de Investigación Sanitaria del Hospital Clínico San Carlos (IdISSC), Madrid, Spain; Medical University of Gdansk, POLAND

## Abstract

**Aim:**

This study aimed to investigate whether different levels of fasting plasma glucose (FPG) and hemoglobin A1c (HbA1c) in prediabetes are associated with hyperfiltration.

**Methods:**

A prospective cohort of 2,022 individuals aged 30–74 years took part in the PREDAPS Study. One cohort of 1,184 participants with prediabetes and another cohort of 838 participants with normal FPG and normal HbA1c were followed for 5 years. Hyperfiltration was defined as an estimated glomerular filtration rate (eGFR) above the age- and gender-specific 95th percentile for healthy control participants, while hypofiltration was defined as an eGFR below the 5th percentile. The prevalence of hyperfiltration was compared for different levels of prediabetes: level 1 of prediabetes: FPG <100 mg/dL plus HbA1c 5.7–6.0% or FPG 100–109 mg/dL plus HbA1c < 5.7%; level 2 of prediabetes: FPG <100 mg/dL plus HbA1c 6.1–6.4% or FPG 100–109 mg/dL plus HbA1c 5.7–6.0% or FPG 110–125 mg/dL plus HbA1c <5.7% and level 3 of prediabetes: FPG 100–109 mg/dL plus HbA1c 6.1–6.4% or FPG 110–125 mg/dL plus HbA1c 5.7–6.4%.

**Results:**

The participants with hyperfiltration were significantly younger, had a higher percentage of active smokers, and lower levels of hemoglobin and less use of ACEIs or ARBs.

Only level 3 prediabetes based on FPG 100–109 mg/dL plus HbA1c 6.1–6.4% or FPG 110–125 mg/dL plus HbA1c 5.7–6.4% had a significantly higher odds ratio (OR) of hyperfiltration (OR 1.69 (1.05–2.74); P < 0.001) compared with no prediabetes (FPG < 100 mg/dL and HbA1c < 5.7%) after adjustment for different factors. The odds ratios for different levels of HbA1c alone in prediabetes increased progressively, but not significantly.

**Conclusions:**

Level 3 of prediabetes based on FPG 100–109 mg/dL plus HbA1c 6.1–6.4% or FPG 110–125 mg/dL plus HbA1c 5.7–6.4% had a significantly higher OR of hyperfiltration compared with participants without prediabetes.

## Introduction

Glomerular hyperfiltration, also called hyperfiltration, is a well-recognized early and reversible stage of kidney damage in subjects with diabetes and hypertension and is a marker for subsequent chronic kidney disease (CKD) progression [1–3].

Previous studies addressing the association between levels of FPG and HbA1c in prediabetes and hyperfiltration have had inconsistent results [4–11]. The inconsistency among studies, use of limited populations, different ethnicities and lack of agreement about an appropriate definition of hyperfiltration may be some of the reasons for the variations in results among studies.

Obesity and the metabolic syndrome (MetS) are also independent risk factors for CKD. The potential mechanisms involve inflammation, lipotoxicity, and hemodynamic effects; and perhaps other, unknown, mechanisms [12]. Moreover, the high metabolic risk is associated with an increased risk of hyperfiltration [13]. Impaired insulin sensitivity may also be involved in the development of renal dysfunction at an early stage, before the prediabetic elevation of glucose or onset of diabetes [14]. Hyperfiltration could be caused by increases in the glomerular capillary plasma flow rate and mean glomerular capillary hydraulic pressure, caused by afferent arteriolar vasodilation and/or by efferent arteriolar vasoconstriction owing to activation of the renin-angiotensin-aldosterone system, thus leading to glomerular hypertension [15–17].

Since hyperfiltration is considered to represent an early and potentially reversible stage of kidney damage, identifying subjects with prediabetes and hyperfiltration might be helpful for implementing preventive and therapeutic strategies in this population.

This study aimed to investigate whether different levels of FPG and HbA1c in prediabetes are associated with hyperfiltration.

## Methods

### Study design and participants

This sub study form part of the PREDAPS study (PREDiabetes en Atención Primaria de Salud) which is a prospective cohort study in which one cohort of 1,184 participants with prediabetes and another cohort of 838 participants with normal fasting glucose (NFG) were followed during 2012 and 2017. A total of 1,453 participants (71.9% of the baseline sample) attended the last follow-up visit: 622 of the cohort with no alterations in glucose metabolism and 831 of the cohort with prediabetes. Baseline data were collected in 2012, in the context of routine clinical practice, by general practitioners distributed across Spain. Participants aged between 30 and 74 years old who consecutively sought medical attention for any reason were invited to participate in the study. In both cohorts, participants were excluded if they had diabetes, terminal disease, pregnancy, surgery, or hospital admission in the previous 3 months at study entry, or any hematologic disease, which could alter HbA1c values. Information about the design and methods of the PREDAPS study have been previously published [18].

Prediabetes was defined as anyone who fulfilled the following criteria: FPG levels between 100 and 125 mg/dL (5,6 and 6,9 mmol/L), and/or a glycated hemoglobin range from 5.7% to 6.4% (39 to 47 mmol/mol) [19]. Patients who had no results in the previous 6 months underwent analysis to determine these values. The study was classified by the Spanish Agency of Medicines and Medical Devices (Agencia Española de Medicamentos y Productos Sanitarios) as a Non-Interventional (Observational) Post-Authorization Study, and the protocol received ethics committee approval by the Parc de Salut Mar Clinical Research Ethics Committee in Barcelona. All the patients included in the study signed the informed consent form required for their participation.

### Measures

For this sub study we used data previously collected during the baseline visit from PREDAPS Study [18]. This data includes, variables related with the age; sex; educational level; family history of diabetes; smoking status; alcohol consumption; physical activity; sedentary lifestyle and diet. Data on blood pressure, height, weight, and waist circumference were obtained by physical examination. Patients were generally considered obese if their body mass index (BMI) was ≥ 30 kg/m^2^ and with central obesity, if waist perimeter was ≥ 102 cm in men and ≥ 88 cm in women. Hypertension was defined if the patient was on antihypertensive drugs or personal history of high blood pressure or had systolic blood pressure (SBP) ≥ 140 mmHg and diastolic blood pressure (DBP) ≥90 mmHg. Laboratory data collected included: FPG and HbA1c; serum hemoglobin; total cholesterol, low-density lipoprotein (LDL) cholesterol, high-density lipoprotein (HDL) cholesterol, non-high-density lipoprotein (non-HDL) cholesterol and triglycerides (TG); serum creatinine, estimated glomerular filtration rate and urinary albumin-to-creatinine ratio (UACR). Hypercholesterolemia was defined as total serum cholesterol ≥ 250 mg/ dL (6,5 mmol/L), low HDL as *<*40 mg/dL (1,0 mmol/L) in men and *<*50 mg/dL (1,3 mmol/L) in women, high LDL ≥ 100 mg/dL (2,2 mmol/L), and hypertriglyceridemia as serum TG ≥ 150 mg/dL (1,7 mmol/L). Metabolic syndrome was defined if the participants met three or more of the following criteria: waist circumference *>*102 cm in men or *>*88 cm in women; TG ≥ 150 mg/dL (1,7 mmol/L); blood pressure ≥130/85 mmHg; HDL *<*40 mg/dL (1,0 mmol/L) in men or *<*50 mg/dL (1,3 mmol/L) in women; and FPG between 110 and 126 mg/dL (6,1 and 7,0 mmol/L) [20].

The adherence to Mediterranean diet was measure according to the study ATTICA and its score published by Panagiotakos [21]. For each twenty types of studied food, subjects have to respond if their consumption was every day, more than three times a week, two times each week, once a week, less than once a week, never or rarely. 0 as a score in each meal is considered as the subject is having a less healthy diet meanwhile 4 is consider the subject as having a very healthy one. The general punctuation of 0 is considered as a low adherence to diet and 80 is considered the maximum one. The adherence to Mediterranean diet was classified in three categories: low (0–53 points), medium (54–59 points) and high (60–80 points). Moreover, subjects were asked about frequency of meals (i.e. at least three meals per day and less than three meals).

Smoking consumption was classified as: smokers, ex-smokers and never smokers. Alcohol consumption was divided in three categories: occasional drinkers, regular drinkers and no-drinkers which includes ex-drinkers and teetotalers. Physical activity was classified according to World Health Organization (WHO) recommendations. Subjects followed the recommendations if they practiced more than 150 minutes per week of moderate aerobic physical activity, more than 75 minutes each week of vigorous aerobic physical activity or an equivalent combination [22].

The eGFR was calculated using the Chronic Kidney Disease Epidemiology Collaboration (CKD-EPI) formula [23]. First, we defined hyperfiltration, normofiltration, and hypofiltration according to the distribution of eGFR in apparently healthy participants (normal FPG and normal HbA1c, normotension and without kidney disease). Hyperfiltration was defined as an eGFR above the age and sex-specific 95th percentile and hypofiltration as an eGFR below the 5th percentile [7]. The distributions of eGFR in participants were divided into 10-year age and sex groups: 30–39, 40–49, 50–59, 60–69 and ≥ 70 years. Urine albumin and creatinine were measured in urine samples from the first-morning void, and UACR ≥ 30 mg/g (3 mg/mmol) was defined as albuminuria. All participants were classified according to eGFR as hyperfiltration, normofiltration, and hypofiltration.

Participants, according to their FPG and HbA1c levels, were classified into three groups: level 1 of prediabetes: FPG <100 mg/dL (5,6 mmol/L) plus HbA1c 5.7–6.0% (39–42 mmol/mol) or FPG 100–109 mg/dL (5,6–6,0 mmol/L) plus HbA1c < 5.7% (39 mmol/mol); level 2 of prediabetes: FPG <100 mg/dL (5,6 mmol/L) plus HbA1c 6.1–6.4% (43–46 mmol/mol) or FPG 100–109 mg/dL (5,6–6,0 mmol/L) plus HbA1c 5.7–6.0% (39–42 mmol/mol) or FPG 110–125 mg/dL (6,1–6,9 mmol/L) plus HbA1c <5.7% (39 mmol/mol) and level 3 of prediabetes: FPG 100–109 mg/dL (5,6–6,0 mmol/L) plus HbA1c 6.1–6.4% (43–46 mmol/mol) or FPG 110–125 mg/dL (6,1–6,9 mmol/L) plus HbA1c 5.7–6.4% (39–46 mmol/mol).

### Statistical analyses

A descriptive analysis was performed describing the baseline characteristics (demographic distribution, lifestyle variables, obesity, hypertension, and biochemical parameters) in participants in each prediabetes group and in participants without prediabetes. Next, we estimated the distribution of these characteristics in the three categories of glomerular filtration (hypofiltration, normofiltration, and hyperfiltration). Data for the continuous variables were presented as means ± standard deviations (SDs) for categorical variables as percentages. Different types of analysis were used for comparison between the groups such as unpaired t-test, variance (ANOVA), or χ^2^ test. Patients with severe chronic kidney disease have not been excluded. Considering values of glomerular filtrate of the basal stage (CDKEPI), we did not have any case with values below 30 (severe kidney failures–G4 and G5-), and only 8 cases with values <45 (moderate-severe kidney failure–G3B).”

We then created a dichotomous variable of glomerular filtration rate—hyperfiltration versus non hyperfiltration- grouping hypofiltration and normofiltration into a single category. The association between levels of FPG and hyperfiltration and between levels of HbA1c and hyperfiltration was measured using logistic regression. We used two consecutive models: the first one (model 1) was adjusted by sex and age; the second one (model 2) was adjusted for the model 1 factors plus body mass index, waist circumference, metabolic syndrome, systolic blood pressure, diastolic blood pressure, alcohol consumption, smoking status, and physical exercise. The measure of association with its 95% confidence interval was the odds ratio, taking as reference the category of participants without prediabetes. All analyses were conducted using the IBM SPSS statistical package.

## Results

**Fig 1:**
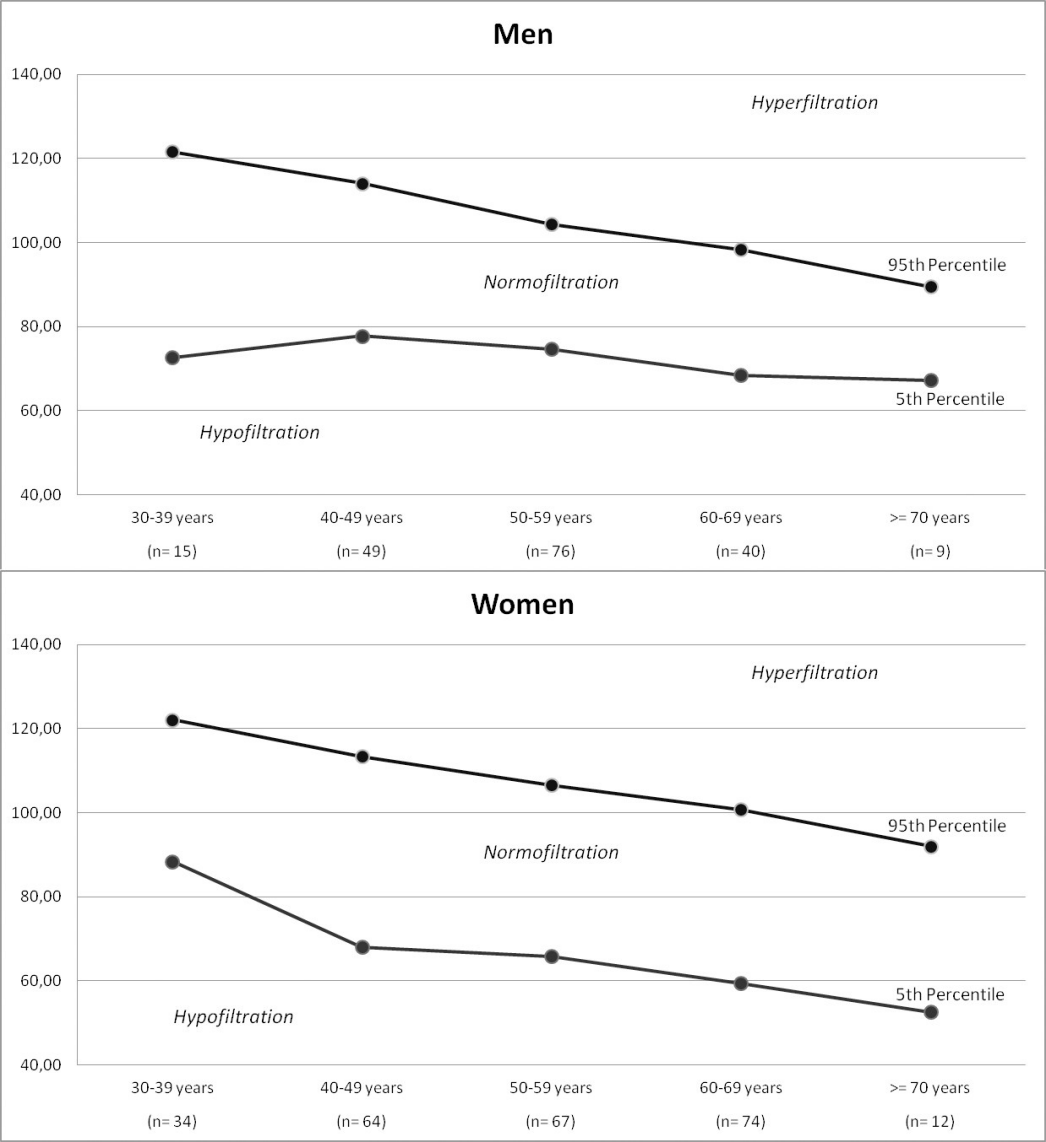
Distribution of estimated glomerular filtration rate (eGFR) in participants with normal fasting glucose and normal HbA1c, normotension and without kidney disease by sex and age groups. The 95th and 5th percentiles are shown in 10-year age groups. Hyperfiltration was defined as an eGFR greater than the age- and gender-specific 95th percentile and hypofiltration was defined as an eGFR less than the 5th percentile.

The study cohort consisted of 2,022 participants (48.6% men). Of these, 1,184 had prediabetes, and 838 had normal FPG and normal HbA1c. Detailed information on the demographic and clinical characteristics of the participants is provided in in S1 Table. The participants belonging to prediabetes level 3 were older and had significantly higher waist circumference, MetS, BMI, FPG, HbA1c, hypertension, SBP, DBP, TG, and more frequent of use of ACE-I/ARB and a smaller percentage of active smokers. Fig 1 shows the distribution of eGFR in participants with normal FPG, normotension, and absence of kidney disease by age and gender.

The prevalence of hyperfiltration was higher in subjects with prediabetes (8.8%) than in subjects with normoglycemia (6.9%), although the difference did not reach statistical significance (p = 0.073).

Table 1 shows the eGFR according to the age and gender of all participants at baseline. The reference values decrease with increasing age, and women have higher eGFR values than men in all age groups in participants with prediabetes, but not in participants with normal FPG and normal HbA1c.

**Table 1:** Estimated glomerular filtration rate (eGFR) according to age and sex of all participants at baseline by glucose metabolism status.

	Prediabetes					Normoglycemia				
	30–39 y N = 39	40–49 y N = 147	50–59 y N = 361	60–69 y N = 473	≥ 70 y N = 162	30–39 y N = 60	40–49 y N = 141	50–59 y N = 257	60–69 y N = 297	≥ 70 y N = 83
eGFR (Total)	106.0 (15.4)	98.2 (13.8)	90.7 (14.7)	83.6 (13.0)	77.7 (13.8)	104.4 (13.7)	97.9 (13.2)	90.9 (11.9)	84.4 (12.9)	75.5 (13.2)
eGFR (Women)	109.2 (12.9)	100.1 (15.2)	92.0 (15.5)	84.5 (12.3)	78.8 (13.9)	104.2 (12.1)	96.5 (14.1)	90.9 (12.6)	84.4 (12.8)	74.1 (13.4)
eGFR (Men)	101.8 (17.7)	96.7 (12.4)	89.3 (13.8)	82.7 (13.6)	76.6 (13.8)	104.9 (16.2)	99.7 (11.9)	91.0 (11.3)	84.4 (12.9)	77.4 (12.8)

Data are mean and (standard deviation) of mL/min per 1.73 m2

Table 2 shows the comparisons of clinical characteristics between participants with hypofiltration (n = 177, 8.8%), normofiltration (n = 1,681; 83.1%) and hyperfiltration (n = 162; 8.1%). The participants with hyperfiltration were significantly younger, had a higher percentage of active smokers, and lower levels of hemoglobin and less use of ACEIs or ARBs. There were no significant differences in other values among the three groups.

**Table 2:** Characteristics of all participants according to filtration at baseline.

Characteristics	Hypofiltration	Normofiltration	Hyperfiltration	p value
	(n = 177)	(n = 1681)	(n = 162)	
Age (years), mean (SD)	60.8 (9.2)	58.1 (9.9)	57.7 (10.2)	0.002
Male, n (%)	128 (72.3)	769 (45.7)	86 (53.1)	<0.001
Smoking status, n (%)				
Active smoker	27 (15.3)	316 (18.8)	41 (25.3)	<0.001
Ex-smoker	89 (50.3)	576 (34.3)	51 (31.5)	
Never smoker	61 (34.5)	789 (46.9)	70 (43.2)	
Regular physical activity, n (%)	120 (67.8)	919 (54.7)	82 (50.6)	0.002
Consumption of alcohol, n (%)	21 (11.9)	202 (12.1)	18 (11.1)	0.931
Adherence Mediterranean diet score, n (%)	79 (44.6)	863 (51.3)	81 (50.0)	0.233
Daily consumption of fruit or vegetables, n (%)	145 (81.9)	1442 (85.8)	137 (84.6)	0.369
Metabolic syndrome, n (%)	60 (33.9)	608 (36.2)	58 (35.8)	0.835
Waist circumference (cm), mean (SD)	99.4 (11.2)	97.0 (12.6)	97.5 (14.1)	0.058
BMI (kg/m^2^), mean (SD)	29.2 (4.2)	28.8 (4.8)	28.9 (5.7)	0.628
Fasting plasma glucose (mg/dL), mean (SD)	99.9 (13.0)	97.4 (13.0)	99.0 (13.4)	0.026
HbA1c (%), mean (SD)	5.6 (0.4)	5.6 (0.4)	5.7 (0.4)	0.390
Hemoglobin (g/dL), n (%)				
≥13.0	160 (90.4)	1492 (88.9)	140 (86.4)	<0.001
12.9–11.0	17 (9.6)	182 (10.8)	16 (9.9)	
≤10.9	0 (0.0)	5 (0.3)	6 (3.7)	
Hypertension, n (%)	121 (68.4)	977 (58.1)	91 (56.2)	0.024
SBP (mmHg), mean (SD)	131.2 (15.1)	132.1 (16.2)	131.2 (15.4)	0.680
DBP (mmHg), mean (SD)	79.5 (9.5)	80.2 (9.5)	79.2 (9.2)	0.281
Total cholesterol (mg/dL), mean (SD)	205.8 (41.0)	210.4 (37.3)	210.9 (38.3)	0.296
HDL-cholesterol (mg/dL), mean (SD)	52.6 (14.5)	56.2 (14.8)	57.1 (16.8)	0.006
Non-HDL-cholesterol (mg/dL), mean (SD)	125.8 (33.5)	129.7 (33.1)	128.7 (33.8)	0.336
Triglycerides (mg/dL), mean (SD)	132.9 (88.9)	124.4 (70.7)	129.0 (85.4)	0.276
Use of ACEIs or ARBs, n (%)	83 (46.9)	529 (31.5)	43 (26.5)	<0.001
Creatinine(mg/dL), mean (SD)	1.2 (0.2)	0.8 (0.1)	0.6 (0.1)	<0.001
eGFR (mL/min per 1.73 m^2^), mean (SD)	61.3 (9.3)	89.3 (12.1)	106.9 (10.9)	<0.001

SBP = systolic blood pressure; DBP = diastolic blood pressure; ACEIs = angiotensin converting enzyme inhibitors; ARBs = angiotensin receptor blockers; eGFR = estimated glomerular filtration rate (CKD-EPI).

Multiple logistic regression analyses for hyperfiltration according to different levels of FPG and HbA1c were analysed. As shown in Table 3, the association between increased FPG and HbA1c and ORs of hyperfiltration was analyzed in three groups. In levels 1 and 2, both FPG and HbA1c levels were not associated with significantly increased ORs of hyperfiltration in model 1 and model 2. In level 3, both FPG and HbA1c levels were not associated with significantly increased ORs of hyperfiltration in model 1, but were associated with significantly increased ORs of hyperfiltration in model 2 after adjustment for age, gender, body mass index, waist circumference, metabolic syndrome, systolic blood pressure, diastolic blood pressure, personal history of arterial hypertension, use of ACEIs or ARBs, drink alcohol consumption, smoking status and physical exercise. The ORs (95% CI) were 1.48 (0.97–2.25) and 1.69 (1.05–2.74) in model 1 and 2 respectively. FPG and HbA1c levels showed a positive linear relationship with an increased odd of hyperfiltration, with level 3 of prediabetes showing significantly increased OR.

**Table 3:** Multiple logistic regression analyses for hyperfiltration according to different level of fasting plasma glucose (FPG) and HbA1c (n = 2005).

Categorical variable of prediabetes	FPG and HbA1c values	Model 1	Model 2
		OR (95% CI)	OR (95% CI)
Non-Prediabetes	FPG <100 mg/dL and HbA1c <5.7%	1.00	1.00
Level 1 Prediabetes	FPG <100 mg/dL plus HbA1c 5.7–6.0% or FPG 100–109 mg/dL plus HbA1c < 5.7%	1.14 (0.72–1.79)	1.19 (0.75–1.89)
Level 2 Prediabetes	FPG <100 mg/dL plus HbA1c 6.1–6.4% or FPG 100–109 mg/dL plus HbA1c5.7–6.0% or FPG 110–125 mg/dL plus HbA1c <5.7%	1.36 (0.86–2.14)	1.52 (0.93–2.48)
Level 3 Prediabetes	FPG 100–109 mg/dL plus HbA1c 6.1–6.4% or FPG 110–125 mg/dL plus HbA1c 5.7–6.4%	1.48 (0.97–2.25)	**1.69 (1.05–2.74)**

Model 1: Sex and age adjusted for baseline.

Model 2: Model 1 plus adjustment for baseline body mass index, waist circumference, metabolic syndrome, systolic blood pressure, diastolic blood pressure, personal history of arterial hypertension, use of ACEIs or ARBs, drink alcohol consumption, smoking status, physical exercise.

Due to physiologic connection between the renin angiotensin system and hyperfiltration, we performed a sensitivity analysis including only 1367 subjects who did not use ACE-I / ARB. The trend of the association at different levels has been maintained, although the magnitude is greater. In prediabetes level 3 the ORs (95% CI) were 1.80 (1.19–2.98) and 2.04 (1.15–3.64) in model 1 and 2 respectively.

## Discussion

In the current study, we examined the relationship between prediabetes and hyperfiltration. After multivariate adjustment, only the odds ratio of hyperfiltration was significantly increased in participants with FPG 100–109 mg/dL (5,6–6,0 mmol/L) plus HbA1c 6.1–6.4% (43–46 mmol/mol) or FPG 110–125 mg/dL (6,1–6,9 mmol/L) plus HbA1c 5.7–6.4% (39–46 mmol/mol). Based on these results, more attention should be paid to participants with FPG and HbA1c in order to maximize the detection and prevention of hyperfiltration in these participants.

It has been reported that hyperfiltration is more prevalent among patients with prediabetes in various ethnic populations. In a Norwegian study [4] of a total of 1,560 individuals aged 50–62 years without diabetes mellitus, an increase in FPG and HbA1c was associated with an elevated risk for hyperfiltration. Participants with impaired fasting glucose (IFG: 100–125 mg/dL) had a multivariable-adjusted odds ratio of 1.56 (95% CI 1.07–2.25) for hyperfiltration compared with individuals with normal FPG. Melsom et al., in another study [5] that included a representative sample of 1,261 persons without diabetes mellitus from the general population, aged 50 to 62 years, found that different measures of borderline hyperglycemia were predictors of higher mGFR and hyperfiltration, but not higher eGFRcr or eGFRcys. According to the authors, this difference may be due to the low precision of the equations used to estimate GFR in the normal GFR range. In a Japanese study [6] of a total of 99,140 people aged 20–89 years, the prevalence of hyperfiltration increased with increasing stage of prediabetes (ORs: 1.29 and 1.58 for FPG 100–109 mg/dL/5,6–6,0 mmol/L and FPG 110–125 mg/dL, respectively; P for trend: <0.001). Okada et al., in their study of a total of 5,003 people aged 35–69 years [7], whose aim was to confirm that hyperfiltration is associated with prediabetes, found that the prevalence of hyperfiltration increased with increasing stages of prediabetes defined by both FPG (fully adjusted ORs: 1.25 and 1.66 for FPG 100–109 mg/dL and FPG 110–125 mg/dL, respectively; p for trend: <0.001) and HbA1c (fully adjusted ORs: 1.26 and 2.12 for HbA1c 5.7–6.0% and HbA1c 6.1–6.4%, respectively; p for trend: <0.001). In a study that included 363 subjects of African descent [8], mean age 44.7 years, 6.6% with IFG, the prevalence of hyperfiltration was 17.2% and 29.2% in NFG and IFG, respectively (p for trend <0.001). Compared to NFG, the adjusted odds ratio for hyperfiltration was 1.99 [95% confidence interval (CI) 0.73–5.44] for IFG. In a Chinese study [9] of 2,491 individuals aged 40–79 years from the general population, HbA1c and FPG were found to be independently and positively associated with hyperfiltration. Sun et al. in their study [10] included subjects ≥20 years of age, and reported that newly diagnosed diabetes and impaired glucose tolerance (IGT), but not isolated IFG, were significantly associated with increased eGFR and a higher risk of hyperfiltration (OR for newly diagnosed diabetes 1.97 (1.48–2.64); p for trend: <0.001; OR for IGT 1.34 (1.07–1.66); p = 0.009; and OR for IFG 1.39 (0.99–1.97); p = 0.06. In a Spanish study [11], 1,281 people from the general population with prediabetes defined as FPG 100–125 mg/dL, aged ≥ 30 years, mean ± SD: 61.4 (13.36) years, had a multivariable-adjusted hazard ratio of 1.52 (1.20–1.91); p for trend <0.001, for hyperfiltration compared with individuals with normal FPG.

In addition to IFG, several parameters of MetS are significant risk factors for hyperfiltration. Different mechanisms are activated in the presence of MetS, leading to hyperfiltration [8,24–26]. If hyperfiltration represents a maladaptive response to metabolic changes [2,26], the process of kidney damage is likely to start early in the course of chronic hyperglycemia. In some studies, hyperfiltration was more closely related to insulin resistance (IR) than to obesity. IR based on homeostatic model assessment (HOMA-IR) was higher in men with hyperfiltration compared to those without hyperfiltration [13,27]. Gender-specific analyses revealed insulin sensitivity (IS) as the main predictor of eGFR in women; whereas 2-hour glucose post oral glucose tolerance test (OGTT) was particularly important in men. Impaired IS, via increasing eGFR and potentially hyperfiltration, may play an independent role in the pathophysiology of renal injury, particularly in females [27]. Another explanation might be the role of high FPG levels, which would stimulate sodium–glucose co-transport in the proximal tubules, thus leading to enhanced proximal sodium reabsorption and diminished distal sodium delivery. Reduced distal sodium delivery, via the tubuloglomerular feedback mechanism, leads in turn to dilatation of the afferent arterioles and hyperfiltration [8,28]. Whole-kidney eGFR is a function of single-nephron eGFR and the total number of nephrons. Nephron numbers vary by gender and birth weight and decrease with age [29]. Because it is not possible to measure single-nephron eGFR directly in living humans, an indirect measure of hyperfiltration based on whole-kidney eGFR must be used in epidemiological studies.

Comparing our data on hyperfiltration with other studies is difficult for several reasons. First, none of these studies has used the gold standard of insulin clearance to measure eGFR. Second, there is no generally accepted cutoff of EGFR to define hyperfiltration, and studies have used different methods to measure eGFR [17,30–31]. Estimated GFR calculated by serum creatinine and/or cystatin C remains the most widely used method in clinical practice and epidemiologic research. A systematic review also indicated that 30% of studies did not justify the choice of threshold values to define hyperfiltration [31]. Fourth, another possible explanation for the discrepancy may be different study designs, such as a relatively small sample size, different age range between the studies, different ethnic populations, or different parameters of MetS, which could lead to varying results. Finally, it is difficult to differentiate between the resolutions of hyperfiltration to the process of nephron loss without performing serial renal biopsies [32]. Finally, we have found in subjects with prediabetes that hyperfiltration was more prominent among women than among men, although the differences in each age group were not statistically significant, probably due to the small sample size. However, in a previous study, a lower GFR decline was noted among men with impaired fasting glucose but not among women [33]. Those conflicting results might be explained by the different methodology of the two studies; our study is a cross-sectional study and the cited study is a historic prospective study.

Our study has several strengths, such as large sample size from a geographically diverse Spanish population with a wide age range. Also, this study adopts the CKD-EPI creatinine-based equation, which is more accurate and has lower bias than the commonly used Modification of Diet in Renal Disease (MDRD) equation, especially at higher levels of GFR [23,34], and hyperfiltration was defined as an eGFR above the age- and sex-specific 95th percentile for apparently healthy participants [7]. On the other hand, this study considers more confounding factors in a fully adjusted model to provide more reliable results. Finally, in a previous study, prediabetes defined by the International Expert Committee shown association with hyperfiltration, whereas prediabetes defined by the American Diabetes Association did not. Our study adds some new valuable information on how this association depends on the levels of FPG and HBA1c, in such a way that the magnitude of the association presents a gradient with the increase in the value of these parameters [35].

However, several limitations warrant mention in this study. First, GFR was estimated rather than using the gold standard of insulin clearance for this measure. Although measured insulin clearance is more accurate than eGFR, it is quite cumbersome, costly and invasive, and is not used in clinical practice in Primary Care. Second, this study design limits inferences on causality. Third, the results cannot be generalized to other ethnic groups since the participants in this study are from the Spanish population, mainly Caucasians. Fourth, in some variables, such as urinary albumin, could not be obtained in a large percentage of subjects (41%) and for this reason this variable was excluded from the analyses. Finally, some participants with normal filtration may have already undergone the hyperfiltration stage.

## Conclusions

Level 3 of prediabetes based on FPG 100–109 mg/dL (5,6–6,0 mmol/L) plus HbA1c 6.1–6.4% (43–46 mmol/mol) or FPG 110–125 mg/dL (6,1–6,9 mmol/L) plus HbA1c 5.7–6.4% (39–46 mmol/mol) had a significantly higher odds ratio of hyperfiltration compared with participants without prediabetes. Our study is a cross-sectional study. Longitudinal studies are needed to confirm whether prediabetes is an independent risk of hyperfiltration and for development of CKD. Furthermore, hyperfiltration should be defined using age- and sex-specific reference values of eGFR and values that reflect the risk of CKD should be established in future investigations. These studies will help to determine whether prediabetes might be a target to prevent kidney damage at an early and reversible stage, and thus prevent the increasing burden of CKD.

## Supporting information

S1 Table Baseline demographic and clinical characteristics. (DOCX)
