# Supplementary material for: High levels of fasting glucose and glycosylated hemoglobin values are associated with hyperfiltration in a Spanish prediabetes cohort. The PREDAPS Study
Source: PLoS One. 2019 Sep 19;14(9):e0222848. doi: 10.1371/journal.pone.0222848 (PMC6752878; doi:10.1371/journal.pone.0222848)
Supplement: S1 Table — (DOCX) [file pone.0222848.s001.docx]

**S1 Table. Demographic and clinical characteristics of all participants at baseline**

|  |  |  |  |  |  |
| --- | --- | --- | --- | --- | --- |
|  | Normoglucose | Level 1 Prediabetes | Level 2 Prediabetes | Level 3 Prediabetes | p value |
|  | (n=838) | (n=400) | (n=346) | (n=438) |  |
| Age (years), mean (SD) | 56.8 (10.4) | 57.9 (9.9) | 59.4 (9.1) | 60.7 (8.8) | <0.001 |
| Male, n (%) | 388 (46.3) | 199 (49.8) | 171 (49.4) | 225 (51.4) | 0.331 |
| Smoking status, n(%) |  |  |  |  |  |
| Active smoker | 187 (22.3) | 76 (19.0) | 54 (15.6) | 67 (15.3) | 0.006 |
| Ex-smoker | 262 (31.3) | 151 (37.8) | 134 (38.7) | 170 (38.8) |  |
| Never smoker | 389 (46.4) | 173 (43.3) | 158 (45.7) | 201 (45.9) |  |
| Regular physical activity, n(%) | 470 (56.1) | 226 (56.8) | 194 (56.1) | 233 (53.2) | 0.714 |
| Consumption of alcohol, n(%) | 89 (10.7) | 47 (11.8) | 51 (14.9) | 54 (12.4) | 0.243 |
| Adherence Mediterranean diet score, n(%) | 400 (47.7) | 202 (50.5) | 199 (57.5) | 223 (50.9) | 0.025 |
| Daily consumption of fruit or vegetables, n(%) | 710 (84.7) | 333 (83.3) | 303 (87.6) | 380 (86.8) | 0.292 |
| Metabolic syndrome, n(%) | 106 (12.6) | 126 (31.5) | 192 (55.5) | 304 (69.4) | <0.001 |
| Waist circumference (cm), mean (SD) | 93.2 (11.9) | 97.0 (12.1) | 100.6 (11.8) | 102.5 (12.4) | <0.001 |
| BMI (kg/m^2^), mean (SD) | 27.4 (4.4) | 28.8 (4.7) | 30.1 (4.7) | 30.7 (5.0) | <0.001 |
| Fasting plasma glucose (mg/dL), mean (SD) | 87.1 (7.3) | 95.9 (7.9) | 105.4 (9.0) | 113.9 (5.8) | <0.001 |
| HbA1c (%), mean (SD) | 5.3 (0.3) | 5.7 (0.3) | 5.8 (0.3) | 6.1 (0.2) | <0.001 |
| Hemoglobin (g/dL), n(%) |  |  |  |  |  |
| ≥13.0 | 722 (86.3) | 365 (91.3) | 314 (90.8) | 392 (89.7) | 0.037 |
| 12.9-11.0 | 110 (13.1) | 31 (7.8) | 32 (9.2) | 42 (9.6) |  |
| ≤10.9 | 5 (0.6) | 4 (1.0) | 0 (0.0) | 3 (0.7) |  |
| Hypertension, n(%) | 398 (47.5) | 233 (58.3) | 232 (67.1) | 328 (74.9) | <0.001 |
| SBP (mmHg), mean (SD) | 128.2 (15.3) | 133.2 (15.9) | 134.6 (17.1) | 135.8 (15.3) | <0.001 |
| DBP (mmHg), mean (SD) | 78.8 (9.6) | 80.7 (9.4) | 80.9 (9.7) | 81.4 (9.0) | <0.001 |
| Total colesterol (mg/dL), mean (SD) | 210.7 (37.5) | 211.2 (37.8) | 207.5 (39.4) | 209.8 (36.6) | 0.520 |
| HDL-cholesterol (mg/dL), mean (SD) | 58.4 (15.5) | 54.5 (14.7) | 54.0 (13.6) | 54.1 (14.7) | <0.001 |
| Non HDL-cholesterol (mg/dL), mean (SD) | 129.5 (32.1) | 131.3 (34.4) | 128.0 (35.8) | 128.1 (31.8) | 0.473 |
| Triglycerides (mg/dL), mean (SD) | 114.7 (74.3) | 129.6 (83.1) | 129.4 (64.7) | 139.1 (66.9) | <0.001 |
| Use of ACEIs or ARBs, n(%) | 205 (24.5) | 120 (30.0) | 130 (37.6) | 200 (45.7) | <0.001 |
| Creatinine(mg/dL), mean (SD) | 0.8 (0.2) | 0.8 (0.2) | 0.8 (0.2) | 0.8 (0.2) | 0.403 |
| eGFR (mL/min per 1.73 m^2^), mean (SD) | 89.2 (14.8) | 87.8 (16.5) | 87.8 (14.1) | 87.0 (15.4) | 0.064 |
|  |  |  |  |  |  |

SBP= systolic blood pressure; DBP= diastolic blood pressure; ACEIs=angiotensin converting enzyme inhibitors; ARBs=angiotensin receptor blockers; NSAIDs: Nonsteroidal antiinflamatory; eGFR=estimated glomerular filtration rate (CKD-EPI).
